# Indole-3-propionic acid inhibits gut dysbiosis and endotoxin leakage to attenuate steatohepatitis in rats

**DOI:** 10.1038/s12276-019-0304-5

**Published:** 2019-09-10

**Authors:** Ze-Hua Zhao, Feng-Zhi Xin, Yaqian Xue, Zhimin Hu, Yamei Han, Fengguang Ma, Da Zhou, Xiao-Lin Liu, Aoyuan Cui, Zhengshuai Liu, Yuxiao Liu, Jing Gao, Qin Pan, Yu Li, Jian-Gao Fan

**Affiliations:** 1 Center for Fatty Liver, Department of Gastroenterology, Xinhua Hospital Affiliated to Shanghai Jiao Tong University School of Medicine, 200092 Shanghai, China; 2 CAS Key Laboratory of Nutrition, Metabolism and Food Safety, Shanghai Institute of Nutrition and Health, Shanghai Institutes for Biological Sciences, University of Chinese Academy of Sciences, Chinese Academy of Sciences, 200031 Shanghai, China; 3 Department of Gastroenterology, Zhongshan Hospital of Fudan University, 200032 Shanghai, China; 4 Department of Gastroenterology, The First Affiliated Hospital of Soochow University, 215006 Suzhou, Jiangsu China; 5 Shanghai Key Lab of Pediatric Gastroenterology and Nutrition, 200092 Shanghai, China

**Keywords:** Non-alcoholic steatohepatitis, Metabolic syndrome

## Abstract

Microbial metabolites have emerged as critical components that mediate the metabolic effects of the gut microbiota. Here, we show that indole-3-propionic acid (IPA), a tryptophan metabolite produced by gut bacteria, is a potent anti-non-alcoholic steatohepatitis (NASH) microbial metabolite. Here, we demonstrate that administration of IPA modulates the microbiota composition in the gut and inhibits microbial dysbiosis in rats fed a high-fat diet. IPA induces the expression of tight junction proteins, such as ZO-1 and Occludin, and maintains intestinal epithelium homeostasis, leading to a reduction in plasma endotoxin levels. Interestingly, IPA inhibits NF-κB signaling and reduces the levels of proinflammatory cytokines, such as TNFα, IL-1β, and IL-6, in response to endotoxin in macrophages to repress hepatic inflammation and liver injury. Moreover, IPA is sufficient to inhibit the expression of fibrogenic and collagen genes and attenuate diet-induced NASH phenotypes. The beneficial effects of IPA on the liver are likely mediated through inhibiting the production of endotoxin in the gut. These findings suggest a protective role of IPA in the control of metabolism and uncover the gut microbiome and liver cross-talk in regulating the intestinal microenvironment and liver pathology via a novel dietary nutrient metabolite. IPA may provide a new therapeutic strategy for treating NASH.

## Fatty liver disease: Bacterial metabolite protects liver by maintaining gut integrity

A molecule made by beneficial microbes in the gut helps protect against a severe form of fatty liver disease by preventing bacterial toxins from leaking out of the intestines into the bloodstream. A research team in China led by Yu Li from the Shanghai Institute of Nutrition and Health (CAS) and Jian-Gao Fan from Xinhua Hospital (SJTU) showed in a rat model of non-alcoholic steatohepatitis that indole-3-propionic acid (IPA), a metabolite produced by gut bacteria, helps maintain the integrity of the intestinal inner lining. The more robust intestinal barrier ensures that bacterial toxins do not enter the bloodstream and eventually reach the liver, where they can trigger injury-inducing inflammation. The findings reveal an interaction between the gut microbiome and the liver, and suggest a role for IPA in the treatment of fatty liver disease.

## Introduction

The human gut harbors trillions of microorganisms, most of which are commensal bacteria, collectively termed the gut microbiota. The gut microbiota is of vital importance in human health and is involved in multiple physiological processes, including development, immune response, and metabolism. Disruption of gut microbiota homeostasis is closely related to the pathogenesis of several diseases, such as autism spectrum disorder^1^, inflammatory bowel disease^2^, and obesity^3^. The influence of the gut microbiota on the host is extensive, yet the mechanism is not fully illuminated. A majority of the effects are believed to be mediated by the metabolites produced by the commensal bacteria utilizing dietary nutrients as precursors. These metabolites are bioactive and have multiple functions. Among them, short-chain fatty acids, which are derived from dietary nondigestible fibers, have been demonstrated to take part in regulating immune reactions^4^ and the metabolic state^5^. Another microbial metabolite, trimethylamine N-oxide (TMAO), promotes cardiovascular disease^6^.

The gut flora can also metabolize dietary tryptophan into indole and its derivatives, such as indole-3-acetic acid (IAA), indoleacrylic acid (IA), indole-3-aldehyde (I3A), and indole-3-propionic acid (IPA). Indole and I3A have been shown to play an important role in maintaining intestinal mucosal homeostasis^7,8^. Likewise, IPA, synthesized by the commensal *Clostridium sporogenes*^9^, is capable of regulating gastrointestinal barrier function via the xenobiotic sensor pregnane X receptor (PXR) and toll-like receptor 4 (TLR4)^10^. Recently, two epidemiologic studies have linked IPA to metabolic disorders. It has been shown that serum IPA levels are negatively correlated with the risk of type 2 diabetes (T2D) and low-grade inflammation, which implies that IPA is a protective factor in T2D^11,12^. However, the role of IPA in metabolic diseases and extraintestinal targets remain to be identified.

Over the past two decades, non-alcoholic fatty liver disease (NAFLD) has emerged as a major public health problem worldwide. It has been estimated that approximately 25% of the world’s population is affected by NAFLD^13^ and that the prevalence will continue to increase^14^. NAFLD is considered to be a hepatic manifestation of metabolic syndrome and is closely related to insulin resistance and genetic predisposition. The spectrum of NAFLD consists of non-alcoholic fatty liver (NAFL), non-alcoholic steatohepatitis (NASH), NASH-related cirrhosis, and hepatocellular carcinoma. NASH is a subtype that can progress to life-threatening situations, such as cirrhosis and hepatocellular carcinoma. Moreover, there is no approved drug regimen to treat NASH for now^15^. Therefore, exploring potential therapeutic strategies for NASH is of great importance and urgently needed.

In this study, we investigated the role of IPA in a rat model of high-fat diet (HFD)-induced steatohepatitis. These in vivo and in vitro studies demonstrate that (1) IPA improves gut dysbiosis and protects against intestinal epithelial barrier damage; (2) endotoxin leakage is reduced in rats in response to IPA treatment; (3) the liver is a target of IPA actions to improve NASH; and (4) endotoxin inhibition in the gut may mediate the beneficial effects of IPA on improving liver function.

## Materials and methods

### Animal model and diets

Male Sprague-Dawley rats at 6 weeks of age were purchased from Shanghai Laboratory Animal Co., Ltd. (Shanghai, China). Rats were fed a standard chow diet or an HFD^16,17^ (fat 33 kcal%, carbohydrates 50 kcal%, protein 17 kcal%, and cholesterol 2%; TrophicDiet, Nantong, China) for 8 weeks. Then, the rats fed an HFD were randomly divided into two groups and treated with IPA (20 mg/kg/day) or vehicle by gavage once daily for 8 weeks. The IPA solution was prepared as previously described^18^. All rats were housed under a 12:12 h light/dark cycle at a controlled temperature. All animal experiment protocols were approved by the Institutional Animal Care and Use Committee of Xinhua hospital affiliated to Shanghai Jiao Tong University School of Medicine (approval No. XHEC-C-2017-220).

### Gut microbiota analysis

Fecal samples were collected immediately upon defecation and stored at −80 °C. Fecal DNA was extracted from fecal samples using a TIANamp Stool DNA Kit (Tiangen, Beijing, China) according to the manufacturer’s protocols. The quality and quantity of DNA was verified with a NanoDrop (Thermo Fisher Scientific, Wilmington, Delaware, USA) and an agarose gel. Extracted DNA was diluted to a concentration of 1 ng/μL and stored at −20 °C until further processing. The V4–V5 region of the bacterial 16S ribosomal RNA gene was amplified by PCR. Amplicons were extracted from 2% agarose gels and purified using an AxyPrep DNA Gel Extraction Kit (Axygen Biosciences, Union City, CA, USA) according to the manufacturer’s instructions and quantified using QuantiFluor™-ST (Promega, Wisconsin, USA). Purified amplicons were pooled at equimolar concentrations and sequenced on an Illumina MiSeq platform (Illumina, San Diego, CA, USA) according to the standard protocols. Raw sequencing data were in FASTQ format. Paired-end reads were then preprocessed using Trimmomatic software^19^ to detect and trim ambiguous bases. After trimming, paired-end reads were assembled using FLASH software^20^. Clean reads were subjected to primer sequence removal and clustering to generate operational taxonomic units (OTUs) using Vsearch software with 97% similarity cutoff^21^. All representative reads were annotated and blasted against the Silva database using RDP classifier (confidence threshold was 70%).

### Serum IPA quantification

Serum levels of IPA were quantified using the UPLC-MS/MS method as previously described^9,10^. Briefly, 20 μL of a serum sample was separated using a column (Agilent Zorbax 300SB-C18). Eluent A was 0.1% (vol/vol) formic acid in water; eluent B was 0.1% (vol/vol) formic acid in acetonitrile (Sigma-Aldrich, St. Louis, MO). The flow rate was 200 μL/min. Then, IPA was quantified using Tandem mass spectrometry. The source parameters in the positive ion mode were as follows: capillary voltage 3500 V, fragmentor voltage 150 V, and skimmer voltage 65 V. A standard curve was made by running IPA standards at concentrations of 0.625, 1.25, 2.5, 5, and 10 μg/mL.

### Hematoxylin and eosin and immunohistochemistry staining

Livers and ilea were fixed in 10% phosphate-buffered formalin acetate at 4 °C overnight and embedded in paraffin wax. Paraffin section (5 μm) were cut and mounted on glass slides for hematoxylin and eosin (H&E) staining as previously described^22^. Immunohistochemistry of liver and ileum sections was performed as previously described^23,24^. Liver sections were incubated with antibodies against myeloperoxidase (MPO) (1:100; Abcam, Cambridge, UK) and F4/80 (1:100; Abcam, Cambridge, UK), and ileum sections were incubated with antibodies against zonula occludens 1 (ZO-1) (1:100; Abcam, Cambridge, UK) and Occludin (1:100; Abcam, Cambridge, UK). Livers embedded in optimum cutting temperature compound (Laborimpex, Brussels, Belgium) were used for oil red O staining for assessment of hepatic steatosis. The procedure was performed as previously described^22^.

### Sirius Red staining and Masson staining

Livers were fixed in 10% phosphate-buffered formalin acetate at 4 °C overnight and embedded in paraffin wax. Paraffin section (5 μm) were cut and mounted on glass slides. Sirius Red staining and Masson staining were performed according to the standard methods in routine pathology. The amount of collagen deposition was quantified by measuring the proportion of Sirius Red-stained and Masson-stained areas, respectively, using color thresholding and measurement of area fraction with ImageJ (National Institutes of Health, Bethesda, MD).

### Histological evaluation

Histological alterations were evaluated based on the SAF score system^25^. Briefly, steatosis was scored from 0 to 3 based on the quantities of large- or medium-sized lipid droplets (0: <5%; 1: 5–33%; 2: 33–66%; 3: >66%). Lobular inflammation was scored from 0 to 2 based on foci of inflammatory cells (0: none; 1: ≤2 foci per 20×; 2: >2 foci per 20×). Ballooning was scored from 0 to 2 (0: normal hepatocytes; 1: presence of clusters of hepatocytes with a rounded shape and pale cytoplasm, usually reticulated; 2: same as 1 with some enlarged hepatocytes, at least twofold the size of normal cells). Fibrosis was scored from 0 to 4 (0: none; 1: perisinusoidal or periportal/portal fibrosis; 2: perisinusoidal and periportal/portal fibrosis; 3: bridging fibrosis; 4: cirrhosis).

### Reagents and antibodies

Indole-3-propionic acid (cat. 220027) and lipopolysaccharide (cat. L2880) were purchased from Sigma-Aldrich (St. Louis, MO). Antibodies against phospho-p65 (cat. 3033), p65 (cat. 8242), phospho-IκBα (cat. 2859), IκBα (cat. 9242), phospho-IKKα/β (cat. 2697), and IKKβ (cat. 8943) were obtained from Cell Signaling Technology (Beverly, MA). Antibodies against ZO-1 (cat. sc-33725), Occludin (cat. sc-133256), and β-actin (cat. sc-69879) and horseradish peroxidase-conjugated anti-mouse, anti-rat, anti-rabbit, and anti-goat secondary antibodies were obtained from Santa Cruz Biotechnology (Santa Cruz, CA).

### Cell treatment

Mouse J774A.1 cells were purchased from the Cell Bank of the Chinese Academy of Sciences (Shanghai, China) and cultured in a humidified incubator at 37 °C and 5% CO_2_. The culture medium was high-glucose DMEM supplemented with 10% heat-inactivated fetal bovine serum, penicillin (200 U/mL), and streptomycin (200 μg/mL). Cells were pretreated with different concentrations of IPA for 1 h, followed by 500 ng/mL lipopolysaccharide treatment for an additional 30 min.

### Immunoblots

Immunoblotting analysis was carried out as described previously^26–28^. In brief, rat liver tissues or cultured cells were homogenized and lysed at 4 °C in lysis buffer (50 mM Tris-HCl, pH 8.0, 1% (v/v) Nonidet P-40, 150 mM NaCl, 5 mM EDTA, 1 mM EGTA, 1 mM sodium orthovanadate, 10 mM sodium fluoride, 1 mM phenylmethylsulfonyl fluoride, 2 μg/mL aprotinin, 5 μg/mL leupeptin, and 1 μg/mL pepstatin). Cell lysates were centrifuged at 14,000 r.p.m. for 10 min at 4 °C, and the resulting supernatant was used for immunoblotting analysis. Protein concentrations in cell lysates were measured using Bio-Rad Protein Assay Dye Reagent. For immunoblotting, 20–50 μg of protein was separated by 8–10% sodium dodecyl sulfate-polyacrylamide gel electrophoresis (SDS-PAGE) and then electrophoretically transferred to a polyvinylidene difluoride membrane in a transfer buffer consisting of 25 mM Tris base, 190 mM glycine, and 20% methanol. The membranes were blocked with 5% nonfat milk in Tris-buffered saline with 0.1% Tween 20 (TBST) and incubated with specific antibodies, followed by incubation with horseradish peroxidase-conjugated secondary antibodies. Immunoblots were visualized by a LumiGLO chemiluminescence detection kit (Cell Signaling Technology). The intensity of bands was quantified using ImageJ (National Institutes of Health, Bethesda, MD).

### RNA isolation and quantitative RT-PCR analysis

Liver tissues were homogenized in TRIzol reagent (Life Technologies, Carlsbad, CA, USA), and total RNAs were reverse transcribed to cDNA using SuperScript II reverse transcriptase (Life Technologies, Carlsbad, CA, USA) and Oligo d(T). The resulting cDNA was subjected to real-time PCR with gene-specific primers in the presence of SYBR Green PCR Master Mix (Applied Biosystems) using the StepOnePlus Real-Time PCR System (Applied Biosystems), as described previously^22^. The following quantitative RT-PCR primer sequences were used: TGTGTCCGTCGTGGATCTGA (forward) and CCTGCTTCACCACCTTCTTGAT (reverse) for mouse GAPDH; CGTCAGCCGATTTGCTATCT (forward) and CGGACTCCGCAAAGTCTAAG (reverse) for mouse TNFα; TTCGTGAATGAGCAGACAGC (forward) and GGTTTCTTGTGACCCTGAGC (reverse) for mouse IL-1β; AGTTGCCTTCTTGGGACTGA (forward) and TCCACGATTTCCCAGAGAAC (reverse) for mouse IL-6; GGGCAGCCCAGAACATCAT (forward) and CCAGTGAGCTTCCCGTTCAG (reverse) for rat GAPDH; TGCCTCAGCCTCTTCTCATT (forward) and GAGCCCATTTGGGAACTTCT (reverse) for rat TNFα; GAAGTCAAGACCAAAGTGG (forward) and TGAAGTCAACTATGTCCCG (reverse) for rat IL-1β; AGTTGCCTTCTTGGGACTGA (forward) and CCTCCGACTTGTGAAGTGGT (reverse) for rat IL-6; AGCCAACTCTCACTGAAGC (forward) and GTGAATGAGTAGCAGCAGGT (reverse) for rat CCL2; CACCGTATGACTATGATGATG (forward) and CAGGAGAGCAGGTCAGAGAT (reverse) for rat CCR2; ATTCCTGGCGTTACCTTGG (forward) and AGCCCTGTATTCCGTCTCCT (reverse) for rat TGFβ; TGTGCTATGTCGCTCTGGAC (forward) and CCAATGAAAGATGGCTGGAA (reverse) for rat αSMA; GGCAGGGCCAACCACTGTGC (forward) and CAGTGCACTTGCCTGGATGG (reverse) for rat CTGF; TGTTCAGCTTTGTGGACCT (forward) and CAGCTGACTTCAGGGATGT (reverse) for rat Col1α1; ACCTCAGGGTGTTCAAGGTG (forward) and CGGATTCCAATAGGACCAGA (reverse) for rat Col1α2; and GGTGGCTTTCAGTTCAGCTATG (forward) and GTCTTGCTCCATTCACCAGTGT (reverse) for rat Col3α1.

### Statistical analysis

Data are expressed as the mean ± SEM. Statistical significance was evaluated using an unpaired two-tailed Student’s *t*-test and among more than two groups by one-way ANOVA. Differences were considered significant at a *P* value <0.05.

## Results

### Gut microbiota dysbiosis in rats fed an HFD is alleviated by the administration of IPA

**Fig. 1:**
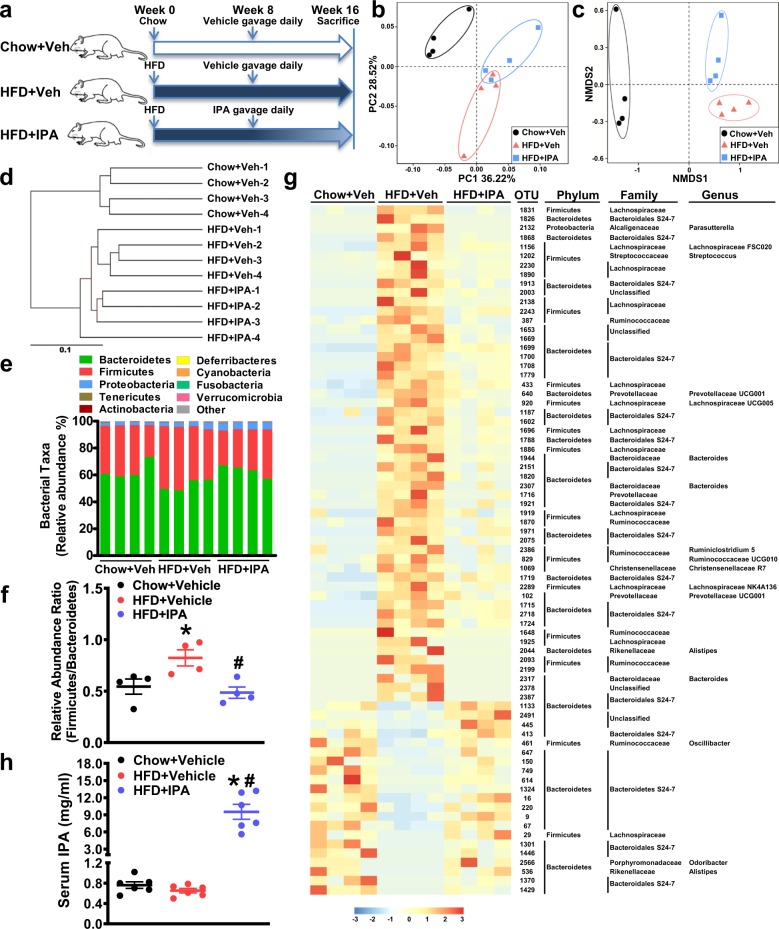
Administration of IPA alleviates gut microbiota dysbiosis in rats fed a high-fat diet. Fecal samples of chow-fed, HFD-fed, and HFD-fed, IPA-treated rats were harvested at week 16. Genomic DNA was extracted from feces, and subsequent 16S rRNA-based gut microbial profiling was performed. **a** Schematic illustration showing the design of the in vivo experiment. **b** Principal coordinate analysis (PCoA) of an unweighted UniFrac distance matrix. **c** Nonmetric multidimensional scaling (NMDS) analysis of a Bray–Curtis distance matrix. **d** UPGMA UniFrac clustering showing sample similarity. Relative abundance of taxa at the phylum level (**e**) and the *Firmicutes* to *Bacteroidetes* ratio (**f**). **g** Heatmap showing the abundance of OTUs significantly altered by the HFD and reversed by IPA treatment. Representative bacterial taxa information (phylum, family, and genus) is shown. **h** The serum levels of IPA were quantified. The data are presented as the mean ± SEM. *n* = 4–6, *P < 0.05, vs. rats fed a chow diet; ^#^*P* < 0.05, vs. rats fed a high-fat diet

To investigate the effects of IPA on the composition of the gut microbiota during nutrient overload, administration of IPA in rats fed an HFD was performed. IPA has shown protective effects against indomethacin-induced intestinal injury; 10, 20, and 40 mg/kg IPA were used to treat mice via gavage, and no obvious side effects were observed^10^. The same doses of IPA (10, 20, and 40 mg/kg) have been used in mice to study the cross-talk between bacterial and mammalian metabolism^9^. Therefore, administration of IPA at 20 mg/kg was performed in rats fed an HFD. Fecal samples were harvested at the end of the study (i.e., week 16) (Fig. 1a), and 16S rRNA-based gut microbial profiling was performed. Principal coordinate analysis (PCoA) and nonmetric multidimensional scaling (NMDS) analysis revealed that HFD feeding caused a major change in the overall composition compared with that in the chow diet-fed group, and 8 weeks of oral IPA administration led to a significant shift in the gut microbial profile compared with that of the HFD + vehicle group (Fig. 1b, c). Cluster analysis showed that samples from the HFD + IPA group differed from those from the HFD + vehicle group (Fig. 1d). Moreover, Adonis and Anosim analyses were performed to assess statistical differences between different groups. The results showed that the composition of the gut bacteria in rats treated with IPA was significantly different from that in rats treated with vehicle (data not shown), suggesting the efficacies of IPA administration on altering the overall structure of the gut bacteria. Then, we assessed the gut microbial profile at the phylum level. An increase in *Firmicutes* abundance and a decrease in *Bacteroidetes* abundance is a hallmark in obesity^29^. In accordance with this feature, we found that HFD feeding caused an increased *Firmicutes* to *Bacteroidetes* ratio and that IPA treatment could reverse it (Fig. 1e, f). Redundancy analysis was applied to identify the specific bacterial phylotypes that were altered by HFD feeding and IPA treatment. A total of 75 OTUs were identified to be altered by HFD feeding and reversed by IPA treatment (54 OTUs increased by HFD and decreased by IPA and 21 OTUs decreased by HFD and increased by IPA) (Fig. 1g). Due to the technological limits of 16S rRNA sequencing, bacterial taxa information at the genus and species level was not fully detailed. Notably, the abundances of two potential pathogenic genera, *Bacteroides* and *Streptococcus*^30^, were increased by HFD feeding and decreased by IPA treatment. The abundance of the *Parasutterella* genus, which is reported to be associated with intestinal chronic inflammation^31^, was also reduced by IPA treatment. Meanwhile, the abundances of *Oscillibacter* and *Odoribacter*, two genera that are implied to be important for intestinal homeostasis^32,33^, were decreased in the HFD + vehicle group and recovered in the HFD + IPA group. Taken together, these results demonstrate that administration of IPA remodels the structure of the gut flora and alleviates dysbiosis induced by HFD feeding.

### Intestinal epithelial barrier damage is diminished by administration of IPA in rats fed an HFD, leading to the inhibition of endotoxin leakage

**Fig. 2:**
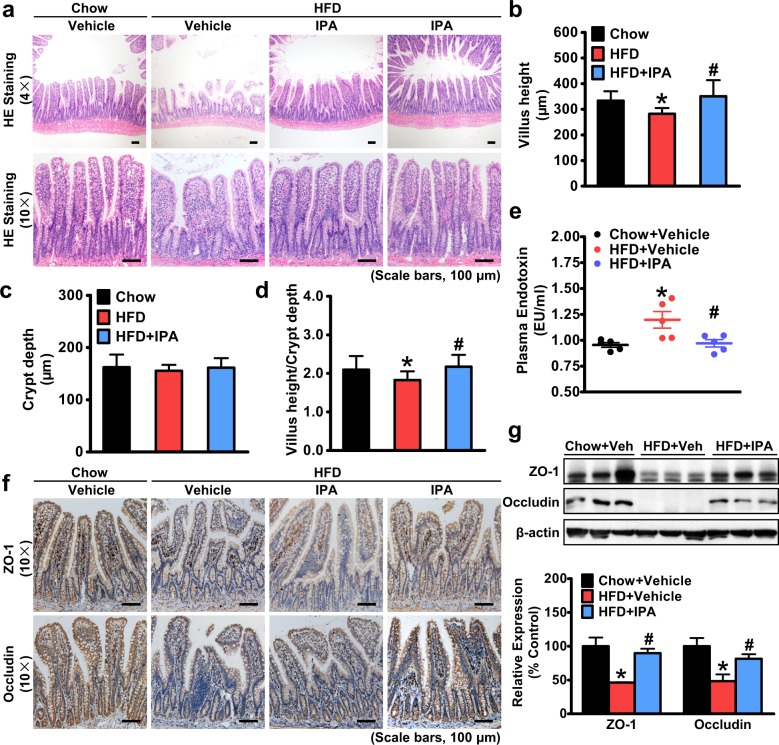
IPA is able to improve intestinal epithelial barrier damage and consequently inhibit endotoxin secretion in rats fed a high-fat diet. Six-week-old male rats were fed a high-fat diet for 8 weeks and then gavaged with IPA (20 mg/kg/day) once daily for 8 weeks. Rats were sacrificed at the end of the 16th week, and ilea were harvested. a Representative hematoxylin and eosin staining of the ilea is shown. The height of the villi (**b**) and the depth of the crypts (**c**) were measured, and the ratio of villus height to crypt depth (**d**) was calculated. **e** The plasma level of endotoxin was measured using a Limulus amebocyte lysate (LAL) chromogenic assay (*n* = 5). **f** Representative immunostaining of zonula occluden-1 (ZO-1) and occludin in the ilea is shown. **g** Protein levels of ZO-1 and occludin of intestinal epithelial cells in the ilea. Representative immunoblots and densitometric quantification from three rats in each group are shown. The data are presented as the mean ± SEM. *n* = 9–10, *P < 0.05, vs. rats fed a chow diet; ^#^*P* < 0.05, vs. rats fed a high-fat diet

Increased intestinal epithelial permeability and small intestinal bacterial overgrowth are frequently observed in several gastrointestinal and metabolic disorders^34^. Thus, we explored whether IPA has a beneficial effect on the epithelial homeostasis of small intestines under HFD feeding conditions. As shown in Fig. 2a, abnormal morphological alterations characterized by a loss of normal villus structure of the ileac epithelium can be observed in rats fed an HFD. Whereas the villus height was decreased, the crypt depth was barely changed, and the villus-to-crypt ratio was decreased in the ilea of HFD-fed rats, and the villus height was restored by IPA treatment (Fig. 2b–d), suggesting that IPA attenuates mucosal lesions caused by HFD feeding.

Furthermore, we examined the expression levels of tight junction proteins, whose loss can lead to increased epithelial permeability, which is also referred to as “leaky gut”. The protein levels of zonula occluden-1 (ZO-1) and Occludin were reduced in the ilea of rats fed an HFD compared to rats fed a chow diet, and IPA treatment upregulated the protein expression of ZO-1 and occludin, as demonstrated by immunostaining and immunoblots (Fig. 2f, g). Moreover, to further examine the effect of IPA treatment on the function of intestinal epithelial barrier, we determined the plasma level of endotoxin. As shown in Fig. 2e, elevated plasma endotoxin levels were observed in the HFD-fed group, while IPA treatment significantly reduced plasma endotoxin levels, suggesting an enhanced intestinal epithelial barrier. These findings indicate a beneficial role of IPA in maintaining intestinal epithelial homeostasis, which results in a reduction in endotoxin leakage from the gut into the bloodstream.

### IPA is sufficient to attenuate hepatic steatosis and restore metabolic homeostasis in rats fed an HFD

**Fig. 3:**
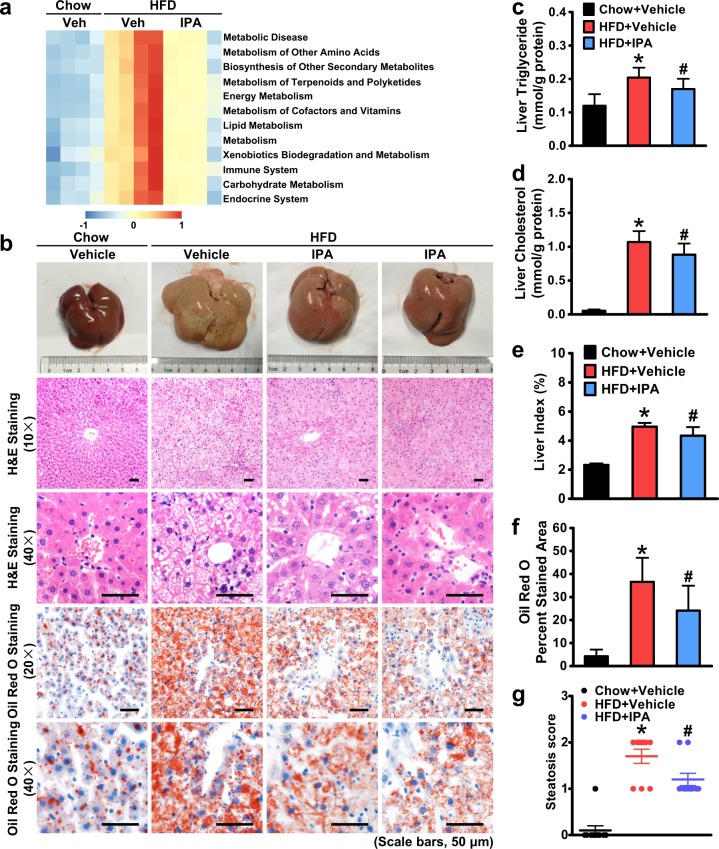
Administration of IPA protects against hepatic steatosis and restores metabolic homeostasis in rats fed a high-fat diet. **a** IPA alters metabolic pathways in the gut microbiota of HFD-fed rats. Prediction of the functional genes in the sampled bacterial community was performed using PICRUSt (*n* = 4). **b** Representative gross pictures of the livers and hematoxylin and eosin and oil red O staining of the liver sections are shown (scale bars, 50 μm). Hepatic triglyceride (**c**) and cholesterol (**d**) levels were assessed in rats. **e** The liver index was calculated. **f** Quantification of oil red O-stained areas is shown. **g** Steatosis score based on the SAF score algorithm was measured. The data are presented as the mean ± SEM. *n* = 7–10, *P < 0.05, vs. rats fed a chow diet; ^#^*P* < 0.05, vs. rats fed a high-fat diet

Hepatic steatosis is a major characteristic in NAFLD. Hence, we examined the effect of IPA on hepatic steatosis induced by HFD feeding. First, we applied the PICRUSt (Phylogenetic Investigation of Communities by Reconstruction of Unobserved States) method to predict functional alterations in the gut microbiota of HFD-fed rats treated with IPA. As shown in Fig. 3a, pathways related to nutrient and energy metabolism were upregulated by the HFD and downregulated by IPA treatment, suggesting that IPA may affect metabolic processes through modulating the gut microbiota. Moreover, LC-MS/MS has been performed to measure serum levels of IPA. As shown in Fig. 1h, a moderate reduction in IPA levels was observed in rats fed an HFD compared with those in rats fed a chow diet. Notably, administration of IPA caused a significant induction of serum levels of IPA, suggesting potential roles of IPA in mediating cross-talk between the gut and extraintestinal organs. Therefore, we explored the effect of IPA on HFD-induced fatty liver. As shown in Fig. 3b, in contrast to control rats, HFD-fed rats developed dramatic liver enlargement and discoloration, which were partially recovered with IPA intervention. Strikingly, HFD-induced lipid droplets in the liver were reduced by IPA treatment, as evidenced by H&E and oil red O staining (Fig. 3b, f). Lipid quantification demonstrated that HFD feeding caused a great induction of lipid accumulation with an increase in triglyceride and cholesterol levels, which were both decreased in the HFD + IPA group (Fig. 3c, d). Correspondingly, the liver index was significantly increased in HFD-fed rats, which was decreased by IPA treatment (Fig. 3e). Moreover, the steatosis score based on the SAF scoring system was applied to evaluate the effect of IPA on hepatic steatosis^25^. As shown in Fig. 3g, IPA could significantly attenuate hepatic steatosis histologically. These results indicate that administration of IPA is sufficient to attenuate HFD-induced hepatic steatosis, possibly through restoration of metabolic homeostasis with modulation of the energy metabolism-related gut microbial profile.

### Hepatic inflammation and liver injury are attenuated by IPA in rats fed an HFD

**Fig. 4:**
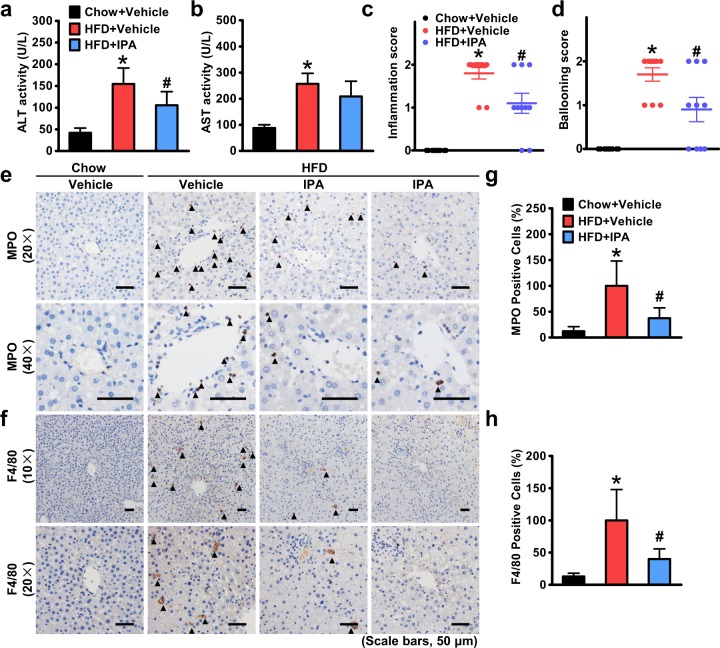
IPA mitigates hepatic inflammation and liver injury in rats fed a high-fat diet. Plasma levels of ALT (**a**) and AST (**b**) were measured. Inflammation (**c**) and hepatocellular ballooning (**d**) were scored based on histological alterations. Representative immunostaining of myeloperoxidase (MPO) (**e**) and F4/80 (**f**) to show infiltration of neutrophils and macrophages, respectively. Arrows denote positively stained cells. Quantification of MPO- (**g**) and F4/80-positive (**h**) cells expressed as a percentage of those in the HFD + vehicle group. The average numbers of positive cells were quantified from a randomly selected pool of five fields under each condition. The data are presented as the mean ± SEM. *n* = 8–10, *P < 0.05, vs. rats fed a chow diet; ^#^*P* < 0.05, vs. rats fed a high-fat diet

Although hepatic steatosis can be a benign lesion, steatohepatitis can progress to cirrhosis, liver failure, and rarely liver cancer and is a recommended indication for pharmacological treatments^35^. To investigate whether IPA has a beneficial role in steatohepatitis, we next assessed the effect of IPA on hepatic inflammation and liver injury induced by HFD feeding. As shown in Fig. 4a, b, plasma levels of alanine transaminase and aspartate aminotransferase were greatly elevated in rats fed an HFD and significantly declined with IPA intervention. Moreover, lobular inflammation and hepatocellular ballooning, which reflect the activity of steatohepatitis, were evaluated based on the SAF scoring system^25^. Strikingly, the inflammation score and ballooning score were increased in HFD-fed rats, and both were decreased by IPA treatment (Fig. 4c, d), suggesting that IPA can reduce steatohepatitis activity histologically. Enhanced hepatic infiltration of inflammatory cells is a key feature of steatohepatitis. Therefore, we examined the infiltration of neutrophils and macrophages using immunostaining of their specific markers. Neutrophils, whose increase coupled with augmented activity of MPO in the liver is closely associated with increased degrees of lobular inflammation in NASH patients^36^, showed increased infiltration in the hepatic lobules of rats fed an HFD, and their infiltration was decreased with IPA intervention (Fig. 4e, g). Similarly, hepatic macrophages, which have been identified as key mediators that trigger inflammatory response during NASH development, were also decreased by IPA treatment compared with those in the HFD + vehicle group (Fig. 4f, h). Taken together, these results indicate that IPA can significantly attenuate HFD-induced hepatic inflammation and liver injury.

### Activity of nuclear factor κB signaling and production of proinflammatory cytokines in response to endotoxin are inhibited by IPA in macrophages

**Fig. 5:**
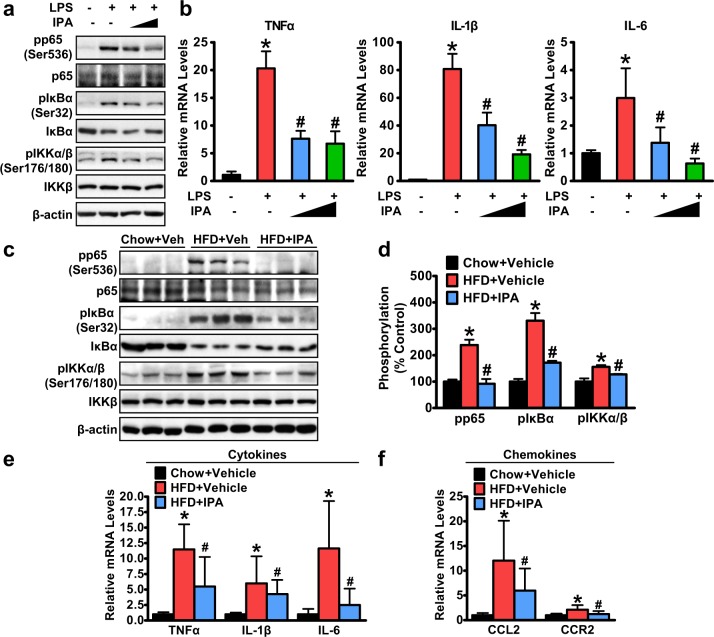
IPA inhibits the activation of NF-κB signaling and the production of proinflammatory cytokines in macrophages in response to endotoxin. **a**, **b** IPA inhibits NF-kB signaling in murine J774A.1 macrophages. Cells were pretreated with IPA (250 μM and 500 μM) for 1 h, followed by 500 ng/mL LPS treatment for an additional 30 min. **a** Representative immunoblots are shown. **b** Expression levels of proinflammatory cytokines were determined by real-time PCR. Relative expression levels were normalized to those of GAPDH. The data are presented as the mean ± SEM (*n* = 4). *P < 0.05 vs. vehicle; ^#^*P* < 0.05 vs. LPS treatment. **c**–**f** IPA inhibits NF-κB signaling in the livers of HFD-fed rats. Representative immunoblots (**c**) and densitometric quantification (**d**) from three rats in each group are shown. Relative phosphorylation levels were normalized to those of endogenous proteins except for phosphorylated IκBα, which was normalized to β-actin. The expression levels of cytokine genes (TNFα, IL-1β, and IL-6) (**e**) and chemokine genes (CCL2 and CCR2) (**f**) were determined by real-time PCR. Relative expression levels were normalized to those of GAPDH. The data are presented as the mean ± SEM. *n* = 7–10, *P < 0.05, vs. rats fed a chow diet; ^#^*P* < 0.05, vs. rats fed a high-fat diet

As hepatic macrophages are exposed to gut-derived LPS that drains into the portal vein and can initiate an immune response and cause subsequent inflammatory injury through the TLR4/NF-κB (nuclear factor κB) pathway^37^, we further assessed whether IPA could directly inhibit inflammatory NF-κB signaling in hepatic macrophages. Murine macrophages were pretreated with IPA and then exposed to LPS stimulation. Strikingly, IPA inhibited LPS-induced phosphorylation of p65 in a dose-dependent manner (Fig. 5a). Upstream signaling of NF-κB was also detected. When exposed to stimuli such as LPS, IκBα, an inhibitor of NF-κB, becomes phosphorylated, resulting in polyubiquitination and proteasomal degradation, which allows free NF-κB to translocate to the nucleus and activate transcription of target genes^38^. Increased phosphorylation and degradation of IκBα were observed in response to LPS stimulation and were significantly suppressed with IPA pretreatment (Fig. 5a). Furthermore, IKKα and IKKβ, two kinases upstream of IκB, were increasingly phosphorylated with LPS stimulation, which was inhibited by IPA pretreatment (Fig. 5a). Moreover, IPA could greatly decrease the expression levels of proinflammatory cytokines, such as tumor necrosis factor-α (TNFα), interleukin-1β (IL-1β), and IL-6, which are NF-κB downstream targets (Fig. 5b), suggesting that IPA can directly suppress LPS-induced activation of NF-κB signaling in vitro.

Furthermore, we determined the effect of IPA administration on the activity of NF-κB signaling in the livers of rats fed an HFD. Strikingly, phosphorylation of p65, IκBα, and IKKα/β was induced with HFD feeding and was significantly inhibited by IPA treatment (Fig. 5c, d). Moreover, we detected the expression levels of cytokines and chemokines in the liver. Prominently, mRNA levels of proinflammatory cytokines, such as TNFα, IL-1β, and IL-6, and chemokines, such as CCL2 and CCR2, were all increased in the livers of HFD-fed rats and were decreased with IPA intervention (Fig. 5e, f). These results indicate that IPA can inhibit the activity of hepatic NF-κB signaling and the production of proinflammatory cytokines, which may contribute to the mitigation of HFD-induced hepatic inflammation and liver injury by IPA treatment.

### Administration of IPA inhibits the expression of genes promoting fibrosis and reduces liver fibrosis in rats treated with IPA

**Fig. 6:**
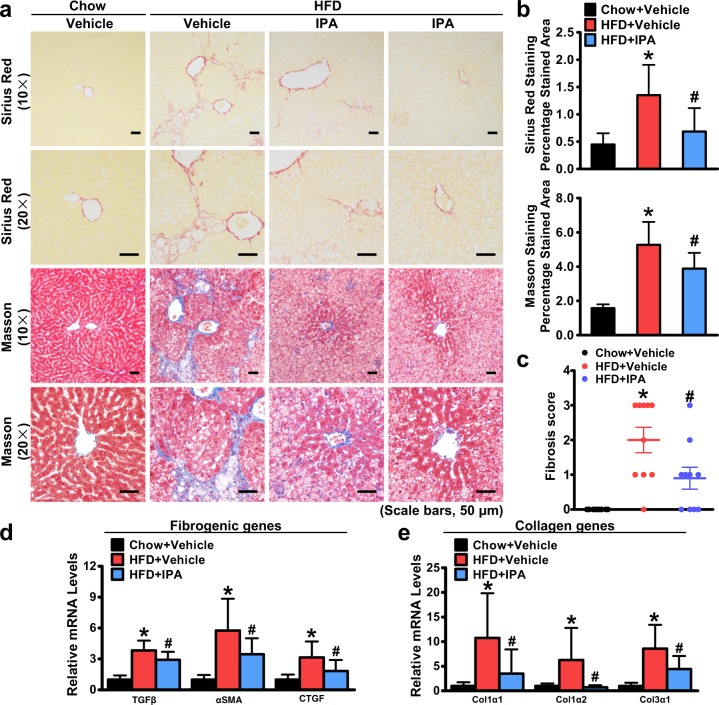
The expression of genes promoting fibrosis is altered in the livers of rats treated with IPA, leading to a reduction in liver fibrosis. **a** Representative Sirius Red and Masson trichrome staining of liver sections (scale bars, 50 μm). **b** Quantification of Sirius Red and Masson-stained areas is shown. **c** Fibrosis was scored based on histological alterations. The expression levels of fibrogenic genes (**d**) and collagen genes (**e**) were determined by real-time PCR. Relative expression levels were normalized to those of GAPDH. The data are presented as the mean ± SEM. *n* = 7–10, *P < 0.05, vs. rats fed a chow diet; ^#^*P* < 0.05, vs. rats fed a high-fat diet

The liver fibrosis stage is the strongest predictor for disease-specific mortality in NAFLD^39^. Therefore, we determined whether IPA administration had an impact on HFD-induced liver fibrosis. As shown in Fig. 6a, b, IPA significantly ameliorated liver fibrosis induced by HFD feeding, as evidenced by Sirius Red staining and Masson trichrome staining. We further examined the effect of IPA on liver fibrosis using the fibrosis score based on the SAF scoring system^25^. The elevated fibrosis score due to HFD feeding was significantly decreased by IPA treatment (Fig. 6c). Moreover, the expression levels of genes related to liver fibrosis were determined. Strikingly, IPA downregulated the expression levels of fibrogenic genes, such as TGFβ, αSMA, and CTGF (Fig. 6d), and collagen synthetic genes, such as Col1α1, Col1α2, and Col3α1 (Fig. 6e). These results suggest that oral administration of IPA for 8 weeks markedly reduces HFD-induced liver fibrosis and prevents the progression of NASH.

## Discussion

Although the tryptophan metabolite IPA has been demonstrated to play a role in maintaining intestinal epithelial homeostasis in an indomethacin-induced intestinal injury model^10^, its other functions and extraintestinal targets remain poorly understood. We demonstrated for the first time that IPA improves gut dysbiosis, protects against intestinal epithelial barrier damage under HFD feeding conditions, reduces endotoxin leakage, and directly inhibits the activity of NF-κB signaling and the production of proinflammatory cytokines, which contribute to the improvement of NASH in HFD-fed rats.

Conventionally, gut microbial metabolites are considered to be mediators in the interaction between the gut microbiota and the host, and the role of microbial metabolites in regulating physiological and pathological processes of the host has been widely studied. However, regulation of the gut microbiota per se by their metabolites has been less focused. Our previous study found that butyrate, a short-chain fatty acid produced by commensal bacteria, can modulate the composition of the gut microbiota and increase the abundance of beneficial *Christensenellaceae*, *Blautia*, and *Lactobacillus*, which in turn increases the production of butyrate, forming a virtuous cycle^23^. Unlike the ability of short-chain fatty acids to decrease gut PH and provide a more suitable intestinal environment for the growth of probiotic bacteria^40^, tryptophan metabolites may function as signaling molecules in interspecies communication of gut commensal bacteria. It has been reported that indole is a nontoxic signal that decreases *E. coli* biofilms by repressing motility, inducing the sensor of the quorum sensing signal autoinducer-1 (SdiA), and influencing acid resistance^41^. Therefore, it is likely that tryptophan metabolites, including IPA, may regulate the composition of the gut microbiota by influencing quorum sensing phenotypes and suppressing virulence factor production. In agreement with this conjecture, we observed that oral administration of IPA could significantly decrease the abundances of pathogenic *Bacteroides* and *Streptococcus*, which are frequently found to be increased in NAFLD patients^42^. These changes likely contribute to the improvement of HFD-induced steatohepatitis with IPA intervention.

Furthermore, the modulation of the gut microbial profile by IPA may benefit the intestinal epithelium, as improved morphology and barrier function were observed in our study. These results are consistent with the recent finding that IPA protects against indomethacin-induced intestinal injury^10^. Apart from the reported mechanisms through xenobiotic receptors, the effect of IPA may also be mediated by the decreased *Parasutterella* and increased *Oscillibacter* and *Odoribacter* abundances, which are closely associated with intestinal epithelial homeostasis^31–33^.

The most important finding of the present study is the identification of cross-talk between the gut microbiota and liver via a novel tryptophan metabolite. IPA is produced by the commensal bacteria in the intestines, which have been shown to be a direct target of IPA^10^. However, as IPA is absorbed by intestinal epithelial cells and diffuses into the bloodstream^9^, IPA can be circulated to the whole body and have multiple targets. In the past two decades, IPA has been identified to function in the brain due to its potent neuroprotective properties as a hydroxyl radical scavenger^43^. However, the association of the serum IPA level with metabolic diseases revealed by epidemiological investigations^11,12^ has provided a cue that the liver may be a target of IPA.

In agreement with the epidemiological investigations, we found that IPA can bona fide protect against HFD-induced steatohepatitis. Herein, we demonstrate that hepatic steatosis induced by HFD feeding is significantly attenuated by IPA intervention. This effect may be due to the regulation of the energy metabolism-related gut microbial profile by IPA treatment. In addition, it is possible that IPA directly participates in the regulation of lipid metabolism. A recent study revealed that another tryptophan metabolite, I3A, suppressed lipogenesis in vitro. I3A treatment significantly reduced the expression of SREBP1c and FAS in murine hepatocytes with or without fatty acid and/or TNFα preconditioning^44^. Considering the structural resemblance of IPA and I3A, they may share functional similarities. In addition to dysregulation of nutrient metabolism, hepatic inflammation is a pivotal characteristic in NASH. Our in vivo results show that IPA treatment can mitigate hepatic inflammation and liver injury induced by HFD feeding. Meanwhile, IPA treatment significantly reduces hepatic expression of proinflammatory cytokines and chemokines, which can lead to alleviation of systemic low-grade inflammation and metabolic dysregulation in multiple organs^45^. The anti-inflammatory property of IPA is consistent with previous observations showing that IPA treatment ameliorates indomethacin-induced intestinal injury^10^ and ampicillin-induced autoimmune encephalomyelitis in mice^46^. These studies support the protective or therapeutic effects of IPA in response to different pathological conditions. Whether IPA treatment affects the production of cytokines during basal conditions requires further investigation. In the current study, administration of IPA at 20 mg/kg was performed, and no obvious side effects were observed. Moreover, an in vitro CCK-8 assay showed that IPA at a dose range of 20–500 μM has no cytotoxicity or inhibitory effects on cell growth in HepG2 cells under basal conditions (data not shown). Further investigation is needed to examine the effects of IPA on rodents under basal conditions and to define the minimum effective doses on NASH. Taken together, our results show that the liver is a target of IPA actions to improve diet-induced hepatic dysfunction.

Although the anti-inflammatory property of tryptophan metabolites has been observed in recent studies, the exact mechanisms are not fully clarified^47^. Our data suggest that IPA can inhibit NF-κB signaling both in vivo and in vitro. On the one hand, in vivo results demonstrate that IPA can restore the intestinal barrier by upregulating tight junction proteins and restrain the leakage of gut-derived endotoxin into the bloodstream, which is an intense stimulus to activate NF-κB signaling via TLR4. Through this mechanism, IPA indirectly inhibits hepatic NF-κB signaling. On the other hand, IPA can directly inhibit LPS-induced activation of NF-κB signaling, as indicated by in vitro studies. The anti-inflammatory effects of IPA in different macrophages require further investigation. Collectively, the dual inhibition of endotoxin by IPA results in the mitigation of inflammatory reactions in the liver.

Endotoxin-mediated TLR4/NF-κB pathway activation in macrophages has been demonstrated to play a pivotal role in the pathogenesis of NASH; depletion of hepatic macrophages or genetic inactivation of TLR4 substantially blunted NASH development in murine models^48^. Our results are in accordance with these previous findings. Inhibition of hepatic NF-κB signaling can significantly attenuate hepatic inflammation and liver injury and decrease the histological activity of NASH. In addition, LPS/TLR4 signaling is shown to participate in metabolic regulation, as LPS/TLR4 inhibition in bone marrow-derived cells improves metabolism and ameliorates diet-induced fatty liver^49^. This finding may partially explain the beneficial role of IPA in HFD-induced hepatic steatosis. Likewise, given that TLR4/NF-κB signaling also mediates the development of liver fibrosis^50^, inhibition of hepatic NF-κB signaling by IPA may underlie the amelioration of liver fibrosis. Based on the above, our findings demonstrate that sabotage of the endotoxin-mediated proinflammatory response by IPA contributes to the remission of NASH.

**Fig. 7:**
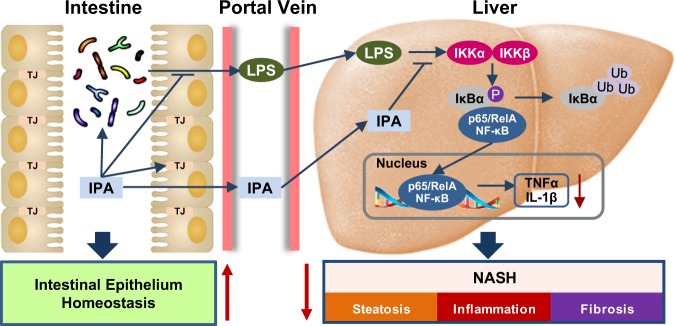
The proposed model for the beneficial effects of IPA on the gut microbiota and steatohepatitis. Administration of IPA improves microbiota dysbiosis, increases tight junction proteins in the gut, and reduces the production of endotoxin. Moreover, IPA attenuates NASH likely through repressing endotoxin-induced activation of NF-κB signaling and production of proinflammatory cytokines, suggesting cross-talk between the gut microbiota and liver

In conclusion, the current study demonstrates that the tryptophan metabolite IPA improves HFD-induced gut dysbiosis and attenuates intestinal epithelial barrier damage, leading to a reduction in endotoxin leakage. Meanwhile, IPA directly inhibits endotoxin-induced activation of NF-κB signaling and production of proinflammatory cytokines (Fig. 7). These functions facilitate IPA’s protection against HFD-induced steatohepatitis in rats. Our results highlight the therapeutic potential of IPA in NASH management.
